# Discovery of new aurone derivatives as submicromolar CK2 inhibitors

**DOI:** 10.1080/14756366.2025.2566780

**Published:** 2025-10-09

**Authors:** Mykhailo V. Mahdysiuk, Galyna P. Volynets, Volodymyr G. Bdzhola, Oleksandr A. Bieda, Sergiy S. Lukashov, Vladislav M. Sapelkin, Leonid L. Karbovskyi, Sergiy M. Yarmoluk

**Affiliations:** ^a^ Institute of Molecular Biology and Genetics, Kyiv, Ukraine; ^b^ Biotech API Laboratory, Kyiv, Ukraine

**Keywords:** Enzyme inhibition, capillary electrophoresis, molecular docking, protein kinase, luminometry

## Abstract

Protein kinase CK2 is a promising therapeutic target, and this study explores 54 aurone derivatives as potential CK2 inhibitors. Activity was evaluated using luminescent and capillary electrophoresis assays, identifying 17 compounds with submicromolar activity. The most potent inhibitors shared key structural features: a benzo group on the A-ring, a hydrogen bond acceptor at the R4′ position, and an additional substituent at the R3′ position of the B-ring. Molecular docking revealed similar binding modes among active compounds, with interactions involving Leu45, Val53, Val66, Met163, Phe113, Lys68, and Ile174. Notably, BFO25 showed the highest activity (IC_50_ = 3 nM at 100 μM ATP). These findings highlight aurones as promising CK2 inhibitors and emphasise the significance of specific structural features.

## Introduction

Protein kinase CK2 is a serine/threonine kinase that regulates numerous essential intracellular processes. Notably, it is involved in several key signalling pathways, including STAT3, PI3K/Akt, TNF-α, and tyrosine kinase receptor pathways^1–4^. Dysregulation of CK2 has been implicated in various diseases, with its role being most studied in a broad spectrum of cancers^5–9^ and viral infections^10–13^. Due to its central role in these pathologies, CK2 has emerged as an attractive therapeutic target.

The use of inhibitors targeting regulatory proteins implicated in various pathologies is well established in clinical practice. Recent advances highlight diverse chemical scaffolds and medicinal chemistry strategies for modulating target proteins, offering potential in anti-inflammatory applications^14–16^. These studies emphasise the importance of continued exploration of novel heterocyclic frameworks as promising sources of new therapeutics.

In recent years, several classes of CK2 inhibitors have been developed, including ATP-competitive, allosteric, and mixed-type inhibitors. The most well-known ATP-competitive inhibitor, CX-4945 (silmitasertib), has entered phase I/II clinical trials for the treatment of cholangiocarcinoma, metastatic basal cell carcinoma, recurrent sonic hedgehog (SHH) medulloblastoma, multiple myeloma, and breast cancer. During the COVID-19 pandemic, silmitasertib was also investigated as a potential therapeutic agent against SARS-CoV-2-induced severe acute respiratory syndrome^17^. Another notable compound, the peptide CIGB-300, demonstrated clinical efficacy in treating squamous intraepithelial lesions and locally advanced cervical cancer^18^.

Previous studies have shown that certain aurone derivatives can inhibit CK2 in micromolar and submicromolar concentrations^19–21^. Investigating new aurone derivatives as CK2 inhibitors can provide deeper insight into the structure-activity relationships of this compound class and potentially lead to the identification of novel therapeutics. The aim of this study was to evaluate 54 newly synthesised aurone derivatives as CK2 inhibitors. This included determining the IC_50_ values of the most active compounds and exploring the potential mechanisms of their interaction with the enzyme through molecular docking.

## Materials and methods

### Chemicals and materials

The study was conducted using recombinant human protein kinase CK2 alpha subunit, obtained using plasmid pZW6 (Addgene, USA, catalogue number 27086), ATP (Sigma-Aldrich, USA, catalogue number A2383) and peptide substrate RRRDDDSDDD (GenScript, USA, catalogue number RP10318). Tris(hydroxymethyl)aminomethane hydrochloride (Tris-HCl) (Sigma-Aldrich, USA, catalogue number 10812846001), KCl (Sigma-Aldrich, USA, catalogue number P3911) and MgCl2 (Sigma-Aldrich, USA, catalogue number 208337) were used to prepare the reaction buffer. Dimethyl sulfoxide (DMSO) (Sigma-Aldrich, USA, catalogue number 472301) was used as a solvent for the compounds. Luminescent assays were performed using Kinase-Glo^®^ kit (Promega, USA, catalogue number V6711). Aurone derivatives were synthesised following the procedure^20^ described earlier starting from the benzo[e]benzofuran-1-one intermediate obtained in a known manner^22^. The structures of tested aurone derivatives are given in the Results section.

### Reaction setup

The reaction mixture volume was 50 μl. The reaction buffer contained 2 mM Tris HCl, 5 mM KCl and 1 mM MgCl2, pH 7.5. The concentration of CK2 alpha subunit was 2 mg/l per sample in control and experimental samples. The reaction mixtures contained 0.5 μl of inhibitor solution in DMSO per sample (final inhibitor concentrations were different in luminescent and CE assays) or 0.5 μl of pure DMSO in control and calibration samples. The samples were incubated at 30 °C for 40 min.

### Luminescent assay

The luminescent assay measured sample luminescence following the addition of the Kinase-Glo luciferase mixture, with the signal reflecting the remaining ATP content. The final ATP concentration indicates the extent of peptide phosphorylation: higher ATP levels correspond to lower peptide phosphorylation. This method was considered as a rapid and straightforward approach for screening a large number of compounds without quantitatively determining their activity.

ATP and peptide substrate were initially present at concentrations of 10 µM and 200 µM, respectively. Calibration samples, which lacked the enzyme, represented the initial ATP concentration. The inhibitor concentration in experimental samples was 1 µM. Measurements were performed using a LuMate 4400 microplate reader (Awareness Technology, USA). Luminescence was recorded after adding 50 µL of the Kinase-Glo luminescent mixture to each sample. In order to simplify the screening process, no signal–concentration calibration curve was constructed for precise ATP quantification. Instead, luminescence values of experimental and control samples were compared directly with those of enzyme-free calibration samples, which reflected the initial ATP level. The percentage of inhibition was determined by the formula:

$$I=100*\frac{\left(M_{e}-M_{c}\right)}{\left(M_{o}-M_{c}\right)}$$

I is the inhibition in percent, M_e_, M_c_ and M_o_ are the luminescence of the experimental, calibration and control samples, respectively. The experiments were performed in triplicate, after which the obtained values were averaged.

Some experimental samples exhibited lower luminescence than the control samples. This effect may result from the inhibition of luciferase activity. The decrease in luminescence reflects not increased ATP consumption by CK2 during peptide phosphorylation, but rather reduced oxyluciferin formation – the luminescent product – caused by luciferase inhibition by the test compound. The manufacturer of the Kinase-Glo kit, as well as practical studies of protein kinases claim the possibility of this effect^23^^,^^24^. This implies that compounds exhibiting such behaviour are unsuitable for screening with this method and may indicate extremely low specificity of inhibition. Furthermore, some compounds with potential luciferase inhibitory activity could have produced false-negative results. However, even if these compounds effectively inhibit CK2, their inhibition of luciferase suggests low specificity, making them unsuitable as promising CK2 inhibitors.

All samples, including those that did not exhibit luciferase inhibition, showed significant variability in inhibition results across different replicates. This deviation was most pronounced in samples with low absolute luminescence values (and consequently, low inhibition). Koresawa and Okabe note that the Kinase-Glo assay may exhibit variability in inhibition between replicates, which is nevertheless acceptable for screening purposes^24^. As noted earlier, the luminescent assay was used solely as a rapid screening tool rather than for quantitative evaluation of compound activity; therefore, the observed deviations do not affect its intended purpose.

### CE assay18

The initial concentrations of ATP and peptide substrate were 100 µM. The reaction was stopped by adding 100 μl of 20 mM EDTA disodium salt. All IC_50_ studies were performed in triplicate. Mean conversion values for each concentration were used for construction of the conversion-versus-concentration curves and IC_50_ determination.

The conversion was determined by the formula:

$$C=100*\frac{P}{P+N}$$

C is the conversion in percent, P is the peak area of the phosphorylated product, and N is the peak area of the substrate. Since previous studies have shown that the phosphorylated product and the unphosphorylated substrate exhibit similar absorption, this method of calculating the conversion is optimal^24^.

Inhibition was calculated using the formula:

$$I=100*\;\frac{C_{o}-C_{e}}{C_{o}}$$

I – inhibition in percent, С_о_ – conversion in control sample, С_е_ – conversion in experimental sample.

The standard deviation of inhibition measurements at each concentration across replicates did not exceed 10%. For the tested compounds, inhibition curves were plotted as a function of concentration and fitted using the Hill equation:

$$y=\frac{x^{n}}{k^{n}+x^{n}}$$

y – inhibition in percent, x – inhibitor concentration, k – IC_50_ value, n – Hill coefficient.

Inhibition curves were built using at least four data points, with additional concentrations tested for some compounds to refine the curve’s shape. The IC_50_ value was determined from the fitted curve as the concentration producing 50% inhibition.

### Electrophoretic separation

All electrophoretic separations were performed with a capillary electrophoresis system Сapel-105M (Lumex). Fused silica (Lumex) of a total length of 75 cm (effective length 65 cm) with inner diameter 50 μm was used. Before use, fused silica was washed for 5 min with 0.1 M HCl solution, then 5 min with distilled water, then 5 min with 0.1 M NaOH solution, again for 5 min with distilled water and for 5 min with background electrolyte (BGE).

A 150 mM orthophosphoric acid (pH 1.2) was used as BGE. Before electrophoretic separation, 350 μl of deionised water was added to the samples. Samples were introduced hydrodynamically at 900 mbar*s. The electrophoresis voltage was 25 kV. Electrophoretic separation was carried out in positive polarity. Detection conducted at a wavelength of 192 nm. Instrument control, data collection and integration were performed with Elforun (Lumex) software.

### Molecular docking

Molecular docking was performed using MzDOCK^25^ to predict the binding mode of aurone derivatives within the ATP-binding site of protein kinase CK2. The docking employed the crystal structure of CK2 in complex with adenylyl imidodiphosphate (PDB ID: 3NSZ)^26^.

## Results

### Luminescent assay

A luminescent assay was used to screen 54 aurone derivatives at a concentration of 1 μM, with the resulting activity values presented in Table 1. Six compounds with inhibition exceeding 80% and deviation within 25% of the result, designated BFO22-27 in Table 1, were subsequently analysed with CE assay to determine their IC_50_ values.

**Table 1.** Structure and inhibitory activity (as % inhibition) of compounds at 1 μM.

№	R4	R5	R6	R7	R2′	R3′	R4′	R5′	I (%)	Compound
1	H	H	H	H	H	F	OH	H	36 ± 12	–
2	H	H	H	H	OH	OCH3	H	H	<25	–
3	H	H	OH	H	OH	H	H	H	<25	–
4	H	H	OH	H	H	H	COOH	H	41 ± 9	–
5	H	H	OH	H	H	Cl	OH	OCH3	48 ± 21	–
6	H	H	OH	H	H	OC2H5	OH	H	<25	–
7	H	H	OCH3	H	OH	H	H	H	<25	–
8	H	H	OCH3	H	H	OH	H	H	<25	–
9	H	H	OCH3	H	H	H	OH	H	<25	–
10	H	H	OCH3	H	H	OCH3	OH	H	27 ± 19	–
11	H	H	OCH2COOH	H	H	F	H	H	<25	–
12	H	H	OCH2COOH	H	H	H	COOH	H	<25	–
13	H	OH	H	H	OH	H	H	H	<25	–
14	H	Br	H	H	H	CH3	OH	H	<25	–
15	H	Br	H	H	H	F	OH	H	<25	–
16	H	Cl	H	H	H	F	OH	H	<25	–
17	H	Cl	H	H	H	CH3	OH	H	<25	–
18	Cl	H	H	Cl	H	CH3	OH	H	<25	–
19	Cl	H	H	Cl	H	F	OH	H	61 ± 15	–
20	CH3	Cl	CH3	H	H	CH3	OH	H	<25	–
21	CH3	Cl	CH3	H	H	F	OH	H	<25	–
22	H	H	C4H4		H	H	COOH	H	<25	–
23	H	H	C4H4		H	H	OH	H	<25	–
24	H	H	C4H4		H	OH	H	H	LI	–
25	H	H	C4H4		H	OH	OH	NO2	LI	–
26	H	H	C4H4		H	OH	NO2	H	LI	–
27	H	H	C4H4		H	CH3	OH	H	LI	–
28	H	H	C4H4		H	OCH3	OH	H	<25	–
29	H	H	C4H4		H	OCH3	OH	Br	LI	–
30	H	H	C4H4		H	OCH3	OH	NO2	LI	–
31	H	H	C4H4		H	COOH	OH	H	<25	–
32	H	H	C4H4		H	Cl	OH	H	68 ± 15	–
33	H	H	C4H4		H	Cl	OH	Cl	<25	–
34	H	H	C4H4		H	Br	OH	H	<25	–
35	H	H	C4H4		H	Br	OH	Br	39 ± 14	–
36	H	H	C4H4		H	F	OH	H	<25	–
37	H	H	C4H4		H	NO2	OH	H	80 ± 13	BFO22
38	C4H4		H	H	H	H	OH	H	<25	–
39	C4H4		H	H	H	H	COOH	H	69 ± 24	–
40	C4H4		H	H	H	OH	H	H	<25	–
41	C4H4		H	H	H	OH	OH	H	<25	–
42	C4H4		H	H	H	OH	OCH3	H	LI	–
43	C4H4		H	H	H	OH	NO2	H	LI	–
44	C4H4		H	H	H	OH	OH	NO2	LI	–
45	C4H4		H	H	H	CH3	OH	H	<25	–
46	C4H4		H	H	H	OCH3	OH	H	85 ± 14	BFO23
47	C4H4		H	H	H	OCH3	OH	NO2	90 ± 7	BFO26
48	C4H4		H	H	H	COOH	OH	H	32 ± 24	–
49	C4H4		H	H	H	F	OH	H	95 ± 23	BFO24
50	C4H4		H	H	H	Cl	OH	H	88 ± 11	BFO25
51	C4H4		H	H	H	Cl	OH	Cl	78 ± 22	–
52	C4H4		H	H	H	Cl	O	OCH3	46 ± 15	–
53	C4H4		H	H	H	Br	OH	H	86 ± 1	BFO27
54	C4H4		H	H	H	Br	OH	Br	69 ± 1	–

*I - inhibition, LI - luciferase inhibition.*

The majority of compounds exhibiting inhibitory activity belonged to the 4,5-benzosubstituted aurone derivatives. Among the 6,7-benzosubstituted derivatives, only BFO22 demonstrated significant activity. In contrast, aurone derivatives lacking benzosubstitution did not display significant inhibitory activity within the submicromolar range. Notably, only the benzosubstituted derivatives also exhibited luciferase inhibition, with the effect most pronounced in the 6,7-benzosubstituted group. This observation highlights the need for further investigation into the specificity of the inhibitory properties within this compound class.

### CE assay

**Figure 1.**
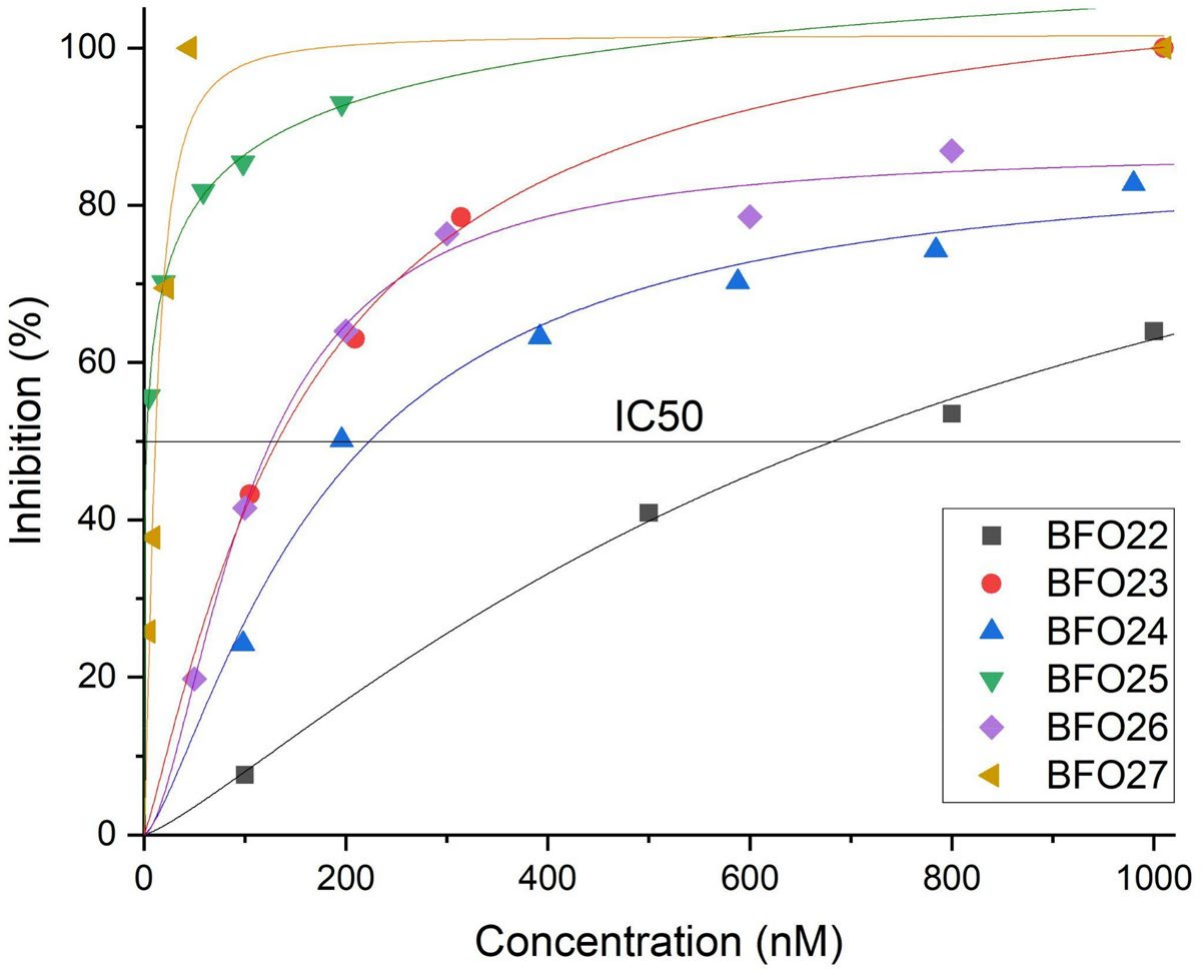
Inhibition-versus-concentration curves.

The IC_50_ values of the compounds selected through the luminescent assay were determined using capillary electrophoresis (CE). The inhibitors were tested at various submicromolar concentrations, enabling the construction of inhibition-versus-concentration curves (Figure 1). All tested compounds exhibited IC_50_ values in the submicromolar range, as summarised in Table 2. It is important to note that, since the ATP concentration in the assay was 100 μM and the compounds act as ATP-competitive inhibitors of CK2, the reported IC_50_ values may be slightly overestimated compared to those obtained using methods with lower ATP concentrations.

**Table 2.** Compound IC_50_ values.

R4	R5	R6	R7	R2′	R3′	R4′	R5′	IC_50_, nM	Compound
H	H	C4H4		H	NO2	OH	H	680	BFO22
C4H4		H	H	H	OCH3	OH	H	130	BFO23
C4H4		H	H	H	OCH3	OH	NO2	130	BFO26
C4H4		H	H	H	F	OH	H	220	BFO24
C4H4		H	H	H	Cl	OH	H	3	BFO25
C4H4		H	H	H	Br	OH	H	13	BFO27

### Molecular docking

**Figure 2.**
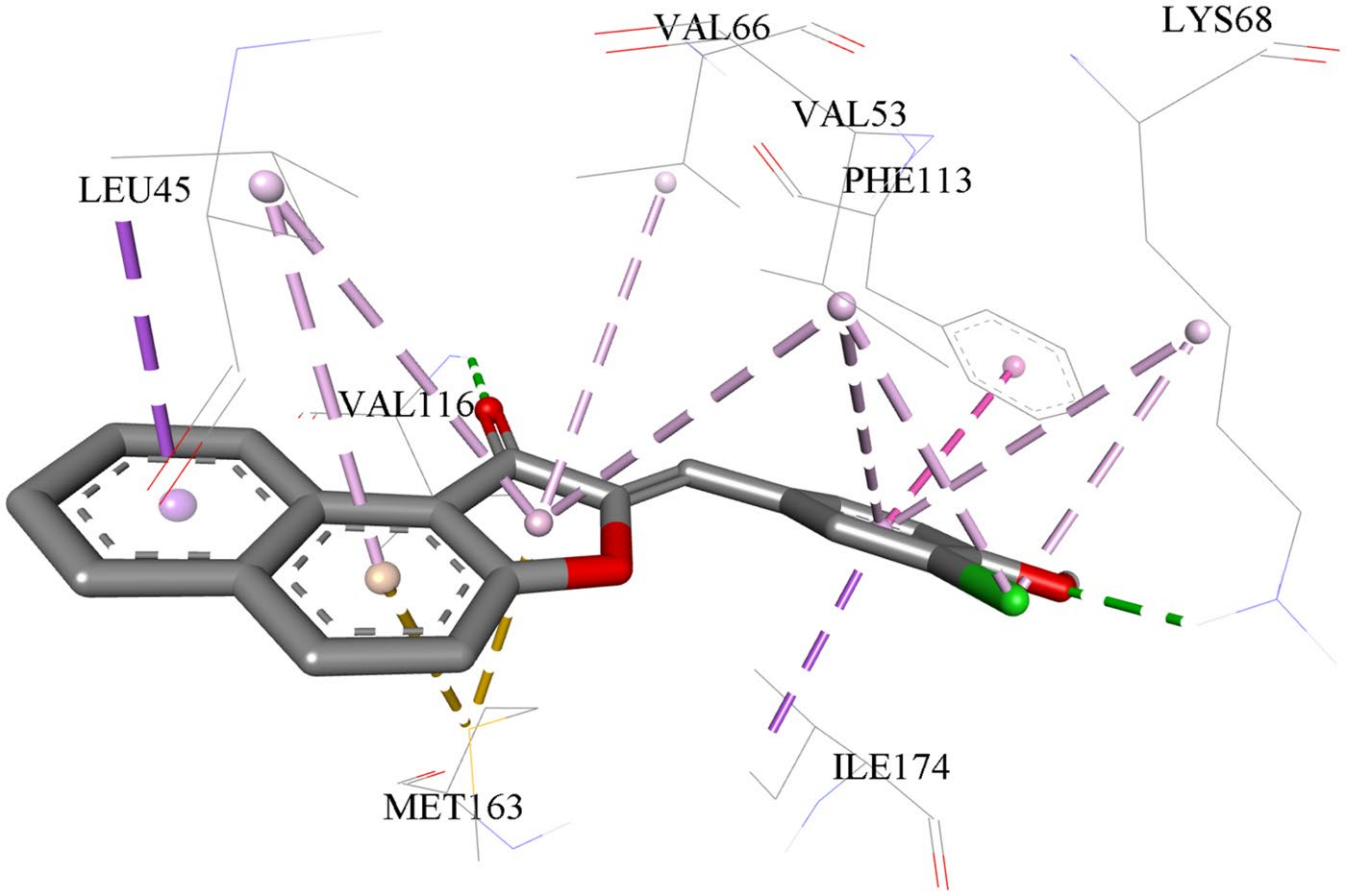
The complex of inhibitor BFO25 with the CK2 ATP-binding site obtained through molecular docking. Hydrogen bonds are indicated in green, hydrophobic interactions in purple, and π–sulfur interactions in yellow.

Figure 2 shows the molecular docking model of inhibitor BFO25 within the CK2 ATP-binding site. The binding mode observed for all active compounds was similar and aligned with previously reported interactions of aurones and flavones^20^^,^^27–30^. The C-ring forms a hydrogen bond with Val116 via its carbonyl group and engages in hydrophobic interactions with Val66, Val53, and Leu45, as well as a π–sulfur interaction with Met163. The A-ring also participates in hydrophobic interactions with Leu45 and forms a π–sulfur interaction with Met163. Notably, the presence of a benzo substituent further enhances the hydrophobic interaction between the A-ring and Leu45. The B-ring interacts hydrophobically with Val53, Phe113, Lys68, and Ile174. Additionally, the hydroxyl group at the R4′ position forms a hydrogen bond with the conserved residue Lys68, while the substituent at the R3′ position engages in interactions with Val53 and Lys68.

## Discussion

### Key structural features of active compounds

The majority of active compounds featured benzosubstitution on the A-ring, predominantly at the 4,5-position. The compound BFO22, which belongs to the 6,7-benzosubstituted group, exhibited the highest (i.e. least favorable) IC_50_ value among the tested active compounds. In contrast, 4,5-benzosubstituted derivatives demonstrated superior inhibitory activity, highlighting their greater potential as a scaffold for the development of novel CK2 inhibitors. Studies suggest that hydrophobic A-ring substituents of flavones play a significant role in CK2 inhibition^20^^,^^27–30^. Molecular docking revealed a hydrophobic interaction between the benzosubstituent and Leu45 (see Figure 2).

Compounds BFO23 and BFO26, both bearing a methoxy group at the R3′ position and differing only by the presence of a nitro group at the R5′ position in BFO26, displayed similar IC_50_ values. This suggests that the introduction of a nitro group at this position does not significantly affect inhibitory potency in this context.

The highest CK2 inhibitory activity was observed for compounds BFO25 and BFO27, which contain halogen substituents at the R3′ position – chlorine and bromine, respectively – with the chlorine-substituted compound showing superior activity. These findings align with the results reported by Protopopov et al., who demonstrated that introducing additional substituents at the R3′ position, adjacent to a hydrogen bond acceptor at R4′, significantly enhances CK2 inhibition. Their structure-activity relationship (SAR) study established the following order of potency for substituents at the R3′ position on ring B: Cl ≥ Br ≥ NO_2_ > OCH_3_ > COOH > H^20^, which is consistent with the experimental data obtained in the present study.

Marzec et al. found that halogenation of natural flavonoids enhances their CK2 inhibitory potential^31^. However, their investigation focused solely on halogen A-ring substitution, without exploring B-ring modifications. In contrast, the current study observed that aurone derivatives bearing halogen A-ring substituents did not exhibit notable activity.

### Structure-activity relationship

Computational analyses, both from previous research and the present study, have identified several key features of the aurone scaffold that are essential for interaction with the CK2 ATP-binding site. One structural element shared with flavonoids is the carbonyl group on the C-ring, which forms a hydrogen bond with Val116. The aromatic rings themselves engage in hydrophobic interactions with Val66, Ile174, and Phe113^20^. Another crucial feature is the hydroxyl group at the R4′ position of the B-ring, which forms a hydrogen bond with Lys68. McCarty et al. reported that flavones active against CK2 consistently possess a hydroxyl group at this position – a characteristic also found in all active aurones identified in this study^32^.

These shared structural features of aurone and flavone scaffolds are fundamental for a compound to act as an ATP-competitive inhibitor of CK2. However, variations in activity among different compounds are determined not by these conserved elements, but by the rings A and B substituents. In particular, the benzo substituent at the R4-R5 positions of the A-ring plays a role in forming a hydrophobic interaction with Leu45, as was already noted.

Functional groups at the R3′-R4′ positions are particularly important, as they interact with the conserved Lys68 residue in the CK2 active site. Molecular docking specifically revealed a hydrophobic interaction of the R3′ halogen substituents and Lys68. The significance of this interaction is further supported by studies on flavones, a compound class structurally related to aurones. The importance of R3′ substituents – particularly the methoxy group – was also demonstrated in the study of the inhibitor FNH79^27^^,^^28^. The hydroxyl groups at R3′-R4′ positions in apigenin and luteolin also participate in hydrogen bonding with CK2, contributing to their inhibitory activity^33^^,^^34^.

## Conclusions

This study identified seventeen novel aurone-based inhibitors of CK2. The inhibitory activity of these compounds was associated with key structural features, notably the presence of a benzo substituent on the A-ring, which contributes through hydrophobic interactions, and a hydrogen bond acceptor at the R4′ position of the B-ring, which forms a hydrogen bond with Lys68. Additionally, the presence of a substituent at the R3′ position further enhanced activity, particularly through interactions with Val53 and Lys68. This effect was most pronounced for halogen substituents such as chlorine and bromine. Overall, the binding mode of the active compounds was consistent with those previously described for aurones and flavones. The most active compound, BFO25, exhibited an IC_50_ of 3 nM at an ATP concentration of 100 μM, highlighting its potential for further investigation. These findings underscore the promise of aurones as CK2 inhibitors and emphasise the critical role of specific structural elements in their mechanism of action.

## Data Availability

The authors confirm that the data supporting the findings of this study are available within the article.
